# Evolution of Public Health Human Papillomavirus Immunization Programs in Canada

**DOI:** 10.3390/curroncol28010097

**Published:** 2021-02-22

**Authors:** Alexandra Goyette, Glorian P. Yen, Voica Racovitan, Parambir Bhangu, Smita Kothari, Eduardo L. Franco

**Affiliations:** 1 Merck Canada Inc., Kirkland, QC H9H 4M7, Canada; voica.racovitan@merck.com (V.R.); parambir.bhangu@merck.com (P.B.); 2 Merck & Co., Inc., Kenilworth, NJ 07033, USA; glorian.persaud.yen@merck.com (G.P.Y.); smita.kothari@merck.com (S.K.); 3 Division of Cancer Epidemiology, McGill University, Montreal, QC H4A 3T2, Canada; eduardo.franco@mcgill.ca

**Keywords:** human papillomavirus, public health programs, Canada, vaccination, vaccine policy, school health services

## Abstract

*Background*: Since 2007, all Canadian provinces and territories have had a publicly funded program for vaccination against human papillomavirus (HPV) infection. The objective of this study was to describe the evolution of these vaccination programs. *Methods*: This was a targeted literature review of public HPV vaccination programs and vaccination coverage rates, based on information provided by jurisdictional public health authorities. *Results:* HPV vaccination of schoolgirls began in school years 2007/08 to 2010/11 with three doses of the quadrivalent HPV vaccine in all provinces except Quebec, which started with two doses. By 2018/19, all jurisdictions were vaccinating with two doses of the nonavalent vaccine in both girls and boys, except Quebec, which used a mixed vaccination schedule with one dose of the nonavalent and one dose of the bivalent vaccines. Public HPV vaccination programs in most provinces include after-school catch-up vaccination. Immunocompromised or other high-risk individuals are eligible for the HPV public vaccination program in most provinces, but policies vary by jurisdiction. In 2017/18, vaccination coverage rates in provincial HPV school-based programs varied from 62% in Ontario to 86% in Prince Edward Island in girls and from 58% in Ontario to 86% in Prince Edward Island in boys. *Conclusions:* Since their introduction, Canadian school-based HPV public vaccination programs have evolved from a three-dose to a two-dose schedule, from a quadrivalent to a nonavalent vaccine, and from a girls-only to a gender-neutral policy. Vaccination coverage rates have varied markedly and only Prince Edward Island and Newfoundland/Labrador have maintained rates exceeding 80%.

## 1. Introduction

Human papillomavirus (HPV) is a sexually transmitted infection [1], with more than 200 genetically distinct types (i.e., strains) of HPV [2]. HPV types have been classified by the International Agency for Research on Cancer as per their carcinogenicity [3]. HPV types 16, 18, 31, 33, 35, 39, 45, 51, 52, 56, 58, and 59 are considered carcinogenic, whereas HPV 68 is considered probably carcinogenic. Several other types are considered possibly carcinogenic, i.e., HPVs 26, 30, 34, 53, 66, 67, 69, 70, 73, 82, 85, and 97 [3]. A few other HPV types, such as 6, 11, 42, 43, and 44, cause benign lesions or asymptomatic infections [2]. Infection with carcinogenic HPV types can lead to cervical, anal, vaginal, vulvar, penile, and oropharynx cancers [1]. Vaccination against human papillomavirus infection could potentially eliminate the most important HPV types that cause cervical, anogenital, and oropharyngeal cancers [4].

Canada was one of the first countries to implement a publicly funded HPV vaccination program. Three vaccines to protect against carcinogenic strains of HPV have been licensed in Canada [5,6]. First, a quadrivalent vaccine (4vHPV) targeting HPV types 6, 11, 16, and 18 was approved in 2006 for use in females 9 to 26 years of age and in 2010 for use in males 9 to 26 years of age. In 2011, the indication was further extended to include women up to 45 years of age. Second, a bivalent vaccine (2vHPV) protecting against types 16 and 18 was approved for females in 2010. Finally, a nonavalent vaccine (9vHPV) targeting carcinogenic types 16, 18, 31, 33, 45, 52, and 58 and low-risk types 6 and 11 was approved for both females and males in 2015.

**Figure 1:**
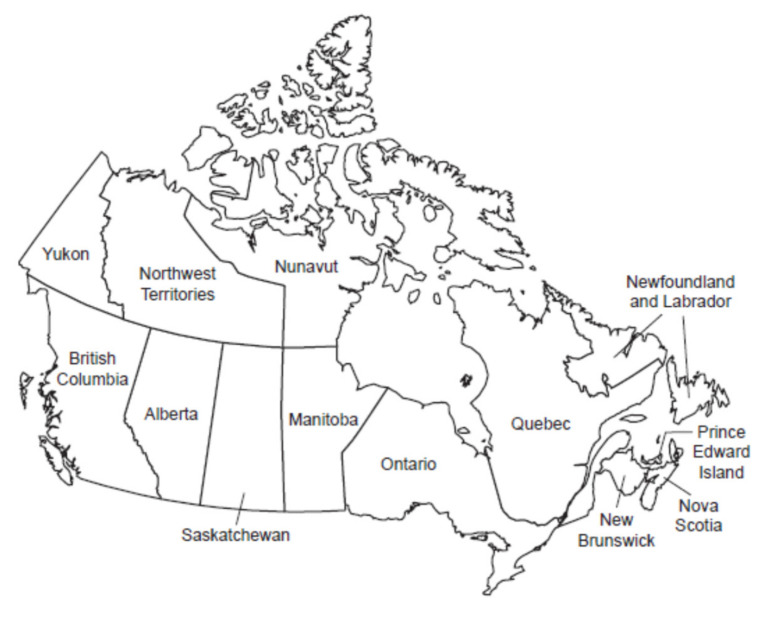
Map of Canada with provinces and territories.

Figure 1 shows the 10 provinces and 3 territories in Canada. Each province and territory is responsible for implementing its public HPV vaccination program. While each jurisdictional program has undergone a series of policy changes, program information is scattered across multiple sources and there is no national database that compiles this information. There have been several descriptions of HPV vaccination programs [5,6,7,8], but no analysis of the evolution of HPV vaccination policies and vaccination coverage in all Canadian jurisdictions. As Canada prepares to implement the prevention strategies designed to meet the World Health Organization’s goal of eliminating cervical cancer [8], it is essential that Canadian policymakers take stock of the current status of HPV vaccination. Hence, the objective of this study is to synthesize publicly available information for each jurisdiction and to describe the evolution of public HPV vaccination programs in Canada.

## 2. Methods

This was a targeted literature review of HPV vaccination programs in Canada, focused on governmental databases and statistics, informal reports, Embase, and PubMed. The scope of the searches was based on the PICO (+) framework (population, interventions, comparisons, outcomes, and time) described in Supplementary Table S1. Searches were conducted between 29 August 2019 and 12 September 2019. We searched the websites of Canadian provincial and territorial health public health authorities with keywords in English and French related to public HPV vaccination programs and vaccination coverage rates. Specific search terms were customized to each province/territory. Examples of search terms are “HPV immunization AND name of province/territory”, “HPV immunization program AND name of province/territory”, “HPV eligibility AND name of province/territory”, “HPV vaccine coverage AND name of province/territory”, “immunization coverage AND name of province/territory”, “immunization coverage report AND name of province/territory”, “immunization report AND name of province/territory”, “immunization coverage AND school AND name of province/territory”, and “vaccine coverage AND name of province/territory”. In addition, we conducted searches of the peer-reviewed literature. PubMed was searched in a heuristic approach, involving chain-searching of related and citing articles. Articles reporting HPV-related studies in Canada were retrieved. Where information was not located using the published literature, local provincial health authorities were directly contacted.

## 3. Data Analysis

We retrieved information by province/territory on the publicly funded, school-based primary grade(s) covered for vaccination, temporary catch-up cohorts, if the programs covered girls and boys, year of program introduction, vaccination schedule, and vaccine coverage rate by year and sex, where published and available. Birth year of children eligible for school-based vaccination programs and other groups eligible for public HPV vaccination programs was also extracted when available. Program details were synthesized by province/territory and year, including vaccination schedule and vaccination coverage rate by sex, and are presented together over time.

All available vaccine coverage rate data were extracted; however, this paper only presents vaccine coverage rates for cohorts that received the full course of HPV vaccination based on the recommended dose regimen at the time of administration.

When not available, birth year of children eligible for school-based vaccination programs was calculated using the assumption that children are five years old when they start kindergarten. For example, if girls and boys are eligible for vaccination in Grade 6 for the 2017/18 school year, it was assumed that they were 11 years old when they were eligible for the first dose of HPV vaccine and it was calculated that the birth year of this cohort was 2006 (2017–11).

Vaccination coverage rate (as a percentage) is expressed as the number of children vaccinated relative to the number eligible for vaccination.

## 4. Ethics Approval

This was a review of publicly available aggregate data and information from published literature; therefore, ethical approval was not required.

## 5. Results

### 5.1. Evolution of Public HPV Vaccination Programs

As shown in Table 1, between school years 2007/08 and 2010/11, all Canadian jurisdictions initiated school-based HPV vaccination for girls within their publicly funded immunization programs. All jurisdictions began with a three-dose schedule (0, 2, and 6 months) of 4vHPV in Grades 6 or 7 (Grades 4–6 in Northwest Territories) except Quebec. In Quebec, the HPV program began with two doses (0 and 6 months) in Grade 4, with a third dose scheduled to be received in Grade 9, five years later. However, in 2013, the program was changed to remove the third dose before the first cohort vaccinated reached Grade 9. In consequence, girls in Quebec have always received two doses, six months apart. Similarly, in British Columbia, the initial three-dose schedule for girls in Grade 6 was changed in 2010 to a two-dose schedule in Grade 6, with a third dose scheduled in Grade 9. The British Columbia program was changed to a two-dose schedule in 2014. Therefore, only the cohort vaccinated in 2010/11 had their third dose in Grade 9; all other cohorts vaccinated after 2010 received two doses. Subsequently, all programs except Alberta transitioned to a two-dose schedule of 4vHPV and then a two-dose schedule of 9vHPV. Alberta was one of the first provinces to transition to 9vHPV vaccine in 2016 and remained at a three-dose 9vHPV scheme until 2018, when the province moved to a two-dose schedule. By the 2018/19 school year, all HPV programs for the 13 Canadian jurisdictions were using a two-dose schedule of the 9vHPV, except Quebec, which adopted in that year a mixed schedule approach with a first dose of 9vHPV followed by 2vHPV for the second dose 6 months later. As of September 2020, Quebec divulged plans to defer the second dose to 5 years later.

In 2013/14, Prince Edward Island was the first province to include boys in the school-based vaccination program. Alberta followed in 2014/15 and Nova Scotia in 2015/16 [9]. All other provinces implemented gender-neutral programs in 2016/17 or 2017/18. By 2018/19, all jurisdictions had gender-neutral, school-based programs with a two-dose schedule of 9vHPV offered in Grades 4 to 7, except that Quebec had a two-dose mixed schedule.

As shown in Table 2, eight of the 13 Canadian jurisdictions instituted a temporary catch-up vaccination program for schoolgirls at either the first or second year of the inception of the program for varying lengths of time (between 1 and 5 years). In 2016, Ontario changed the target age of its vaccination program from Grade 8 to 7 and added one additional cohort so girls in Grade 8 would not be missed during the transition. Alberta, Manitoba, and Quebec were the only provinces that implemented temporary catch-up programs lasting 3 or 4 years for schoolboys at the first or at the second year post program inception. Table 3 shows the birth years of children that have been eligible for the school-based HPV vaccination programs since the introduction of the programs up to the end of the 2019/20 school year.

In addition to the school-based programs, in most of the jurisdictions, individuals who missed the full course while at school remained eligible, as shown in Table 4. Finally, most jurisdictions have public HPV vaccination programs for high-risk groups, which comprise men who have sex with men, transsexual persons, HIV-infected individuals, and other at-risk groups (Table 4).

### 5.2. HPV Vaccination Coverage Rates in School-Based Programs

**Figure 2:**
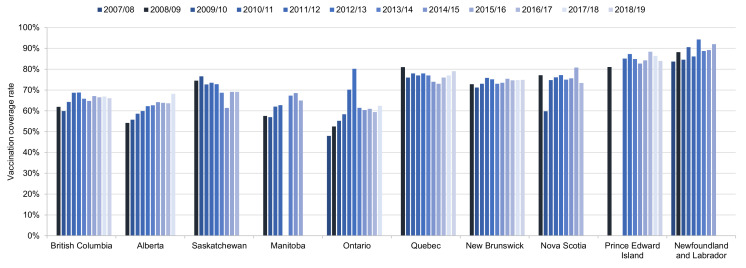
Time trends in HPV vaccination coverage rates in schoolgirls in school years 2007/08 to 2018/19 in the 10 provinces with data. Vaccination coverage rates are reported for full course of treatment at the time of the vaccination. Vaccination coverage rates may not be comparable across provinces as each province used its own methodology. Source: Canadian Partnership Against Cancer System Performance and provincial health authorities [10,12,13,15,17,19,20,21,31,37,38,39].

Time trends in HPV vaccination coverage rates (full course) in schoolgirls from 2007/08 to 2018/19 are shown in Figure 2. Rates in girls ranged from 48% in Ontario in the first year of the program to 94% in Newfoundland/Labrador in 2012/13. Only Prince Edward Island had rates consistently over 80%: in girls in years 2011/12 to 2018/19 and in boys in 2014/15 to 2018/19. Rates were over 80% in Nova Scotia in boys in 2015/16 and 2016/17. For some provinces (Alberta, Ontario), there is evidence that rates gradually increased over the first several years of the program, while in Newfoundland/Labrador, rates improved consistently over the entire time period. In Saskatchewan, rates in girls declined gradually over the time period, from 75% in 2008/09 to 69% in 2016/17. There is no obvious time trend in the remaining provinces.

**Figure 3:**
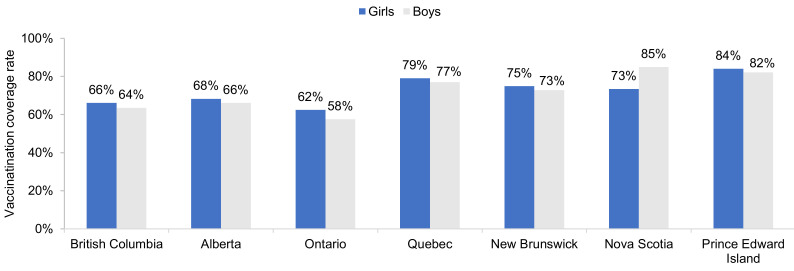
Most recent HPV vaccination coverage rates in schoolgirls and schoolboys, by province. Shown are the most recent data only for provinces with vaccination of both girls and boys. Data are for 2016/17 for Nova Scotia. Data are for 2017/18 for Alberta and Ontario. Data are for 2018/19 for British Columbia, Quebec, New Brunswick, and Prince Edward Island. Source: Canadian Partnership Against Cancer System Performance and provincial health authorities [10,11,12,13,14,15,16,17,18,19,20,23,36,37,39].

The most recent HPV vaccination coverage rates (in 2016/17, 2017/18, or 2018/19) in school children are shown in Figure 3. The highest rates occur in Prince Edward Island in girls (84%) and boys (82%), and Nova Scotia (boys only, 85%).

## 6. Discussion

Since their introduction in 2007, Canadian publicly funded HPV vaccination programs have evolved generally from a three-dose to a two-dose schedule, from a quadrivalent to a nonavalent vaccine, and from a girls-only to a gender-neutral program. HPV programs have been shown to be effective, resulting in decline in the incidence of pre-cancerous cervical lesions and genital warts in Canada [40].

In addition to the school-based programs, in most jurisdictions, individuals who missed the full course while at school remained eligible for a certain period of time to receive publicly funded HPV vaccine. Research has shown that providing opportunities to receive missed doses in schools through catch-up programs is important in optimizing coverage [6].

Most jurisdictions have enhanced their public HPV vaccination programs to include high-risk groups, such as men who have sex with men, transsexual persons, HIV-infected individuals, and other at-risk groups. However, there is no standardization and programs vary across provinces and territories.

The transition to a mixed HPV vaccine dose schedule in Quebec in the fall of 2018 has been widely debated, stemming from the limited clinical efficacy data of this schedule before adoption [41,42,43,44]. To date, no other jurisdiction in the world has adopted a mixed dose schedule [45]. Following COVID-19 disruptions to school-based vaccine delivery in September 2020, the schedule was further modified to include a deferral of the second dose to 5 years after the first dose. In the absence of long-term durability and/or effectiveness data, the real-world impact of this schedule on HPV-related outcomes in these cohorts may not be fully understood for at least a decade [41].

The current COVID-19 pandemic has disrupted many immunization activities across Canada. School closings, originated from the need to control viral transmission, and disruptions from competing priorities in the capacity of the public health system have led to a decrease in coverage in the school-based programs. Provincial surveillance mechanisms are currently assessing the extent of the vaccination delays and losses in coverage [45].

Despite the challenges imposed by its large land area (the second largest country in the world), sparsely populated rural areas, and lack of a central coordinating authority for overseeing immunization delivery, Canadian HPV vaccination programs have largely succeeded, relative to other high-income countries. However, current vaccine coverage rates fall short of the Canadian Partnership Against Cancer (CPAC)’s target to eliminate cervical cancer by 2040. CPAC’s recommendations include attaining: 90% of 17-year-olds to be fully vaccinated by 2025; 90% of eligible women to have been screened with an HPV test by 2030; and 90% of all women with an abnormal screening result to receive within 3 months appropriate diagnostic and treatment by 2030 [46].

Vaccination coverage rates have varied markedly by jurisdiction, and there have been no consistent time trends. Irrespective of jurisdiction, coverage rates in schoolboys have been similar to those in schoolgirls. Since the 2012/13 school year, only Nova Scotia, Prince Edward Island, and Newfoundland/Labrador have had vaccination coverage rates exceeding 80% in any school year. The recommendations of the Canadian Immunization Committee, that 80% of eligible schoolgirls receive the required doses of HPV vaccine within 2 years and 90% within 5 years of program introduction, have largely not been met [47]. More recently, the public health national immunization goal is to achieve by 2025 90% vaccination coverage (two or more doses of HPV vaccine) by 17 years of age in all adolescents [48].

Provinces with the same vaccination program evolution—e.g., Saskatchewan, Ontario, New Brunswick, and Prince Edward Island—have widely different coverage rates. There is no clear evidence that a change in the number of doses was associated with changes in vaccination coverage. There are eight jurisdictions that switched from a three-dose to a two-dose strategy and in only two of them was there a more than five percent increase in vaccination coverage: Saskatchewan and Nova Scotia, both in 2014/2015 to 2016/2017. Similarly, switching the vaccine (but maintaining the same number of doses) had no apparent effect on vaccination coverage. There were six instances of switching the vaccine while maintaining a two-dose schedule and in no instance was there a change in vaccination coverage of ≥ 5%.

Gilbert et al. (2016) explored determinants of HPV non-vaccination and vaccine refusal in girls 12–14 years old, based on data from the Childhood National Immunization Coverage Survey of 2013 [49]. Among sociodemographic variables, a younger age (12 years) at vaccination and a European country of birth of the responding parent were associated with an increased risk of not being vaccinated, while parental knowledge, attitudes, and beliefs were associated with parental refusal of HPV vaccination [49].

A 2014 study of a nationally representative sample of Canadian parents of boys aged 9–16 examined psychosocial factors in parents’ HPV vaccine decision-making for their son [50,51]. Grounded in the Precaution Adoption Process Model, results from the online survey illustrated that discussion with a healthcare provider about the HPV vaccine, higher HPV knowledge, increased perception of risks in the absence of HPV vaccination or that others endorse HPV vaccination, and positive attitudes related to vaccines in general were associated with increased odds of being in the more advanced stages of the decision to vaccinate their son. Believing that HPV vaccination is harmful increased the odds of deciding not to vaccinate while perceiving the benefits of HPV vaccination decreased the odds [50].

More recently, Tatar et al. (2019) examined parental HPV vaccine hesitancy from a nationally representative sample of Canadian parents of 9–16 years old boys and girls in 2016 and 2017 [52]. HPV-related attitudes, behaviors, knowledge, and intentions to vaccinate changed over time in parents categorized as “flexible” hesitant (unengaged/undecided) compared to “rigid” hesitant (decided not). Higher social influence, HPV knowledge, and family income, white race, and lower perception of harms (vaccine safety) were associated with higher HPV vaccine acceptability in “flexible” hesitant parents. Given that healthcare in Canada is managed by the individual provinces and territories, there is substantial variation in the delivery and administration of the HPV vaccine across the 13 different programs [6].

Vaccine coverage rates and type of service delivery model may also be impacted by the diverse geography and contextual means of delivery across the Canadian landscape. In Calgary, Alberta, during the period 2008–11, the HPV vaccine was offered free of charge to all girls in Grades 5 and 9, with two different service delivery models, depending upon the acceptance of the program by the local school board—immunization against HPV was not permitted in-school in the Calgary Catholic School District and at a small number of private schools, and in these cases, vaccination was provided in the community at Public Health Clinics [53]. HPV vaccination completion rates were much higher for girls with an in-school model than for girls with a community service model (75% versus 36%) [53,54].

Bird et al. (2017) reported a meta-analysis of vaccine uptake rates in six of the Canadian provinces based on 12 studies published in the period 2010–2016 [55]. Rates of vaccine uptake varied by age, sex, service delivery model, and funding source. Rates in those ≤18 versus >18 years old were 67% versus 14%, in female versus male (57% versus 47%), in school-based versus community-based programs (69% versus 19%), and with public funding versus out-of-pocket payment (67% versus 14%).

Many countries in Europe and the Americas included HPV vaccination of adolescents in their national immunization programs, beginning in 2007 [56]. These programs differ in service delivery method (school- or clinic-based), public funding, inclusion of catch-up vaccination, etc. [56]. However, programs in some countries are similar to those in Canada. For instance, Australia implemented publicly funded, school-based HPV vaccination in 2007, targeting females 12–13 years of age, with catch-up vaccination, and extending the program in 2013 to include males [57]. As of 2015, estimated coverage with three doses was 77% for females and 66% for males [57]. Scotland initiated a similar program, but without including males, and achieved a three-dose coverage rate in the target cohort (12–13-year-old females) of 90% [58]. HPV vaccination programs in most countries, however, are clinic-based [56]. Notably, the United States initiated clinic-based HPV vaccination in 2006, targeting females 11–12 years, and adding males 11–12 years in 2011 [57]. As of 2015, estimated coverage with at least one dose was only 42% among females and 10% among males 19–26 years of age [58].

## 7. Limitations

The present study is limited in that there are gaps in the available information. Not all HPV vaccine coverage rates have been reported for all years, age, and sex by each jurisdiction in Canada. Coverage in other eligible groups outside school-based is rarely available. Canada does not have a national vaccination surveillance program; therefore, there is a lack of standardized methodologies for reporting vaccination coverage rates across jurisdictions, making it difficult to compare vaccination rates [6,59]. We reported the vaccine coverage rates as provided by provincial health authorities and did not investigate the methodology employed by each individual health unit.

## 8. Conclusions

In conclusion, since their implementation in schoolgirls in 2007, provincial and territorial programs have undergone multiple policy changes, gradually increasing population protection via inclusion of boys and high-risk groups. Over time, policy changes have reflected the evolving science of HPV vaccination and funding support. While there are methodological variations across jurisdictions in how data are collected and reported, HPV vaccination rate targets clearly remain to be met in the school-based public programs in Canada. The data collated in this report may assist Canadian health authorities in addressing the shortfall in HPV vaccination coverage, with the eventual goal of eliminating HPV-related cervical and other cancers.

## Figures and Tables

**Table 1:** Evolution of school-based public HPV vaccination programs and vaccination coverage rates.

Province/territory	School Grade of Vaccination		School year											
			2007/08	2008/09	2009/10	2010/11	2011/12	2012/13	2013/14	2014/15	2015/16	2016/17	2017/18	2018/19
British Columbia ^a^	Grade 6	Girls		61.9%	59.9%	64.3%	68.7%	68.8%	65.8%	64.8%	67.1%	66.5%	66.9%	66.1%
		Boys											64.6%	63.5%
Alberta ^b^	Grade 6	Girls		54.2%	55.7%	58.6%	59.9%	62.2%	62.6%	64.2%	63.9%	63.6%	68.2%	N/A
		Boys								60.3%	62.9%	63.6%	66.1%	N/A
Saskatchewan ^c^	Grade 6	Girls		74.5%	76.6%	72.7%	73.5%	72.8%	68.7%	61.4%	69.1%	69.1%	N/A	N/A
		Boys											N/A	N/A
Manitoba ^d^	Grade 6	Girls		57.6%	57.0%	62.0%	62.7%	N/A	67.3%	68.5%	65.0%	N/A	N/A	N/A
		Boys										N/A	N/A	N/A
Ontario ^e^	Grade 7	Girls	48.0%	52.5%	55.2%	58.4%	70.2%	80.2%	61.5%	60.4%	61.0%	59.4%	62.4%	N/A
		Boys										53.4%	57.5%	N/A
Quebec ^f^	Grade 4	Girls		81.0%	76.0%	78.0%	77.0%	78.0%	77.0%	74.0%	73.0%	76.0%	77.0%	79.0%
		Boys										72.0%	74.0%	77.0%
New Brunswick	Grade 7	Girls		72.8%	71.2%	73.0%	75.8%	75.1%	73.0%	73.5%	75.4%	74.7%	74.8%	74.9%
		Boys											70.2%	72.8%
Nova Scotia ^g^	Grade 7	Girls	N/A	77.1%	59.8%	74.8%	76.1%	77.2%	75.0%	75.6%	80.8%	73.4%	N/A	N/A
		Boys									81.0%	84.9%	N/A	N/A
Prince Edward Island	Grade 6	Girls	N/A	81.1%	N/A	N/A	85.1%	87.3%	84.9%	82.7%	84.3%	88.4%	86.4%	84.0%
		Boys							79.0%	81.4%	85.0%	89.7%	85.6%	82.1%
Newfoundland/Labrador	Grade 6	Girls	83.7%	88.2%	84.6%	90.6%	86.1%	94.3%	88.7%	89.2%	92.0%	N/A	N/A	N/A
		Boys											N/A	N/A
Yukon	Grade 6	Girls			N/A	N/A	N/A	N/A	N/A	N/A	N/A	66.5%	N/A	N/A
		Boys											N/A	N/A
Northwest Territories ^h^	Grades 4–6	Girls			N/A	N/A	N/A	N/A	39.3%	N/A	55.0%	N/A	N/A	N/A
		Boys											N/A	N/A
Nunavut ^i^	Grade 6	Girls				N/A	N/A	N/A	N/A	N/A	N/A	N/A	N/A	N/A
		Boys											N/A	N/A

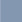
 Three doses 4vHPV; 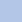
Two doses 4vHPV; 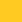
Three doses 9vHPV; 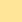
Two doses 9vHPV;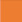 9vHPV + 2vHPV; N/A, not available. ^a^ In 2010, the British Columbia program schedule changed. Girls in Grade 6 in 2010/11 were vaccinated with two doses and received their 3rd and final dose in Grade 9. Girls in Grade 6 in 2011/12 to 2013/14 were scheduled to receive their 3^rd^ dose in Grade 9, but the HPV program changed to two doses in 2014 and, therefore, these girls never received their 3rd dose. ^b^ In 2018, the Alberta program schedule changed from Grade 5 to Grade 6. Vaccination was in Grade 5 from 2008/09 to 2017/2018 and moved to Grade 6 in 2018/19. In 2018, the cohort in Grade 6 had already received the vaccine in Grade 5; therefore, no primary cohort was vaccinated for the school year 2018/19. Of note, Alberta reports coverage rates based on school year grade only since 2017/18 school year. Therefore, from 2008/09 to 2016/17, coverage rates were calculated based on 3 doses by age of 12; coverage rate might not be comparable year to year. ^c^ In Saskatchewan, vaccination coverage rates are calculated based on age cohorts and not on school grades. Therefore, the coverage rate of a given cohort is available only once they reach a certain age (i.e., 13, 15, or 17 years old). For example, for the cohort vaccinated in Grade 6 in 2016/17, the coverage rate is only available once they reach 13 years old, two years later, in 2018 (assuming they were aged of 11 years old at the time of the vaccine). For cohorts vaccinated in 2008/09 and 2009/10, calculations were based on 3 doses by the age of 15 as these were the only rates available for those cohorts. For cohorts vaccinated in 2010/11 to 2014/15, calculations were based on 3 doses by the age of 13, and for cohorts vaccinated in 2015/16 and 2016/17, calculations were based on 2 doses by the age of 13. ^d^ In Manitoba, vaccination coverage rates are calculated based on age cohorts (i.e., 13 and 17 years old). For cohorts vaccinated from 2008/09 to 2011/12, vaccination coverage rates were calculated at age 17, and for cohorts vaccinated from 2013/14 to 2015/16, vaccination coverage rates were calculated at age 13. ^e^ In 2016, the Ontario program schedule changed. Vaccination was in Grade 8 from 2007/08 to 2015/16 for females and moved to Grade 7 in 2016/17. As of 2018/19, the coverage rate reported by Ontario combined girls and boys and was 57.9%. ^f^ From 2008/09 to 2012/13, in Quebec, girls in Grade 4 received two doses of the 4vHPV and were scheduled to receive their 3rd dose in Grade 9. The program changed to two-dose in 2013 and, therefore, girls in Quebec never received the 3rd dose. In 2018, Quebec moved from 2 doses of the 9vHPV vaccine to a mixed vaccination schedule with one dose of the 9vHPV vaccine and one dose of the 2vHPV vaccine ^g^ In 2009/10, the Nova Scotia Public Health department was responding to the H1N1 flu pandemic and resources were only available to conduct immunization clinics for one grade level. Therefore, in the 2009/10 school year, Grade 10 students were immunized, and in 2010/11, Grades 7 and 8 were immunized. ^h^ Although Northwest Territories vaccinate girls in Grades 4–6, coverage estimates are conducted in Grade 7. Assume that vaccination was switched to two-dose vaccination schedule in 2016/17 based on Merck & Co., Inc., Kenilworth, NJ, USA, internal data. Some individuals may have received three doses. ^i^ Nunavut program was implemented in March 2010. Assume that vaccination was switched to two-dose vaccination schedule in 2016/17 based on Merck & Co., Inc., Kenilworth, NJ, USA, internal data. Some individuals may have received three doses. Sources: Provincial health authorities [10,11,12,13,14,15,16,17,18,19,20,21,22,23,24,25,26].

**Table 2:** School-based temporary catch-up HPV vaccination programs.

Province/Territory	Cohorts that Were Eligible for School-Based Catch-Up HPV Program	
	Girls	Boys
British Columbia	Catch-up in Grade 9 from 2008/09 to 2010/11	No catch-up program
Alberta	Catch-up in Grade 9 from 2009/10 to 2011/12	Catch-up in Grade 9 from 2014/15 to 2017/18
Saskatchewan	Catch-up in Grade 7 in 2008/09	No catch-up program
Manitoba	No catch-up program	Catch-up in Grades 8 or 9 from 2016/17 to 2018/19
Ontario	Catch-up in Grade 8 in 2016/17 ^a^	No catch-up program
Quebec	Catch-up in Grade 9 from 2008/09 to 2012/13	Catch-up in Grade 9 from 2018/19 to 2020/21
New Brunswick	Catch-up in Grade 8 in 2008/09 Catch-up in Grade 8 in 2010/11 ^b^	No catch-up program
Nova Scotia ^c^	Catch-up in Grade 10 in 2009/10 Catch-up Grade 8 in 2010/11	No catch-up program
Prince Edward Island	No catch-up program	No catch-up program
Newfoundland/Labrador	Catch-up in Grade 9 from 2008/09 to 2009/10	No catch-up program
Yukon	Catch-up in Grades 7 and 8 from 2009/10	No catch-up program
Northwest Territories ^d^	Catch-up in Grades 11 and 12 in 2009/10 Catch-up in Grades 10 and 11 in 2010/11 Catch-up in Grades 9 and 10 in 2011/12 Catch-up in Grade 9 from 2012/13 to 2014/15	No catch-up program
Nunavut	No catch-up program	No catch-up program

^a^ In the 2016/17 school year, the program moved from Grade 8 to Grade 7. Girls in Grade 8 were offered the HPV vaccine so that this cohort would not be missed during the transition. ^b^ In the 2009/10 school year, the HPV vaccine was delayed in some areas of the province because of the H1N1 mass immunization campaign. During the following school year, 2010/11, the HPV vaccine was offered to female students in Grade 8, where delays occurred because of the H1N1 campaign. ^c^ In 2009/10, the Nova Scotia Public Health service was responding to the H1N1 flu pandemic and resources were only available to conduct immunization clinics for one grade level. A catch-up program was already in place in Grade 10 for other vaccines. Therefore, HPV vaccine was offered to girls in Grade 10. The following year, both Grade 7 (primary cohort) and Grade 8 were immunized (catch-up cohort). ^d^ Information for Northwest Territories was based on the Canada Communicable Disease Report and the information could not be confirmed with officials from the Northwest Territories. However, as of 2017, the province covers HPV vaccination for individuals aged 9–26. Sources: National Advisory Committee and provincial health authorities [17,18,27,28,29,30].

**Table 3:** Birth years of children that have been eligible for school-based HPV vaccination programs (as of end of 2019/20 school year).

Province/Territory	Birth Year																		
	1992	1993	1994	1995	1996	1997	1998	1999	2000	2001	2002	2003	2004	2005	2006	2007	2008	2009	2010
British Columbia																			
Alberta																			
Saskatchewan																			
Manitoba																			
Ontario																			
Quebec																			
New Brunswick																			
Nova Scotia																			
Prince Edward Island																			
Newfoundland/Labrador																			
Yukon																			
Northwest Territories																			
Nunavut																			

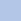
 Girls only birth cohort; 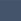 Girls and boys birth cohort; 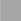 Temporary catch-up cohort ^a a^ In Quebec, there is currently a temporary catch-up cohort in place and Grade 9 boys will be vaccinated in 2020/21. Sources: Provincial health authorities [10,11,12,13,14,15,16,17,18,28,30,31].

**Table 4:** Other groups eligible for public HPV vaccination programs.

	Catch-Up Out of School	MSM	Transgender	HIV	Other High-Risk
British Colombia	Females born ≥ 1994 and males born ≥ 2006 who did not get the vaccine in Grade 6 remain eligible if they start their vaccine series before their 19th birthday and complete it before their 26th birthday.	Males aged 9–26 years who: have sex with other men or are not yet sexually active but are questioning their sexual orientation.	Transgender individuals aged 9–26 years.	HIV positive individuals aged 9–26 years.	Males aged 9–26 years who are street involved. Males aged 9–18 in the care of the Ministry of Children and Family Development. Males of any age who are in youth custody services centres.
Alberta	Females and males who were eligible in Grade 6 are eligible up to the age of 26.	Males aged 17–26 years.			Hematopoietic Stem Cell Transplant recipients between the ages of 9 years up to the end of Grade 12. Solid Organ Transplant candidates and recipients between the ages of 9–26 years.
Saskatchewan	Females born ≥1994 and males born ≥2006 are eligible up to the age of 26.			Individuals aged 9–26 years.	Immunocompromised individuals aged 9–26 years.
Manitoba	Females born ≥1997 and males born ≥2002.	Males aged 9–26 years who identify as gay or bisexual.	Transgender individuals aged 9–26 years.	Immunocompetent HIV-infected females aged 9–45 years. Immunocompetent HIV-infected males aged 9–26 years.	Females aged 9–45 years and males aged 9–26 years who have congenital or acquired immune deficiencies. Males aged ≤ 18 years who are, or who have ever been, incarcerated.Individuals with recurrent respiratory papillomatosis. Females aged 9–45 years who have a newly diagnosed high-grade cervical histopathology result. Females aged 9–45 years and males aged 9–26 years who are victims of sexual assault. Patients currently under the care of a hematologist or oncologist who have malignant neoplasms or have completed immunosuppressive therapy or hypo- or asplenic.
Ontario	Females and males are eligible to initiate or complete the series until the end of their Grade 12 year, if previously eligible for the Grade 7 or 8 programs.	Males ≤ 26 years.	Individuals ≤ 26 years.		
Quebec	Females < 18 years at their first dose. Males who have completed Grade 4 since 2016/17.	Males ≤ 26 years.		Individuals ≤ 26 years.	Males aged 9–17 years who attend rehabilitation centres for youth in difficulty, who are under the care of youth protection services, or who are homeless. Individuals ≤ 26 years who have weakened immune system.
New Brunswick	Females born ≥ 1995 and males born ≥ 2005 are eligible up to the age of 26.				
Nova Scotia	Females and males who have missed or refused HPV vaccine as part of the school-based program (beginning September 2015 in male) up to and including 18 years of age.	Males ≤ 45 years.		Individuals ≤ 45 years.	
Prince Edward Island	Females and males are eligible if missed HPV immunization in Grade 6 (since 2007 for females and 2012 for males).	All eligible individuals regardless of age.		All eligible individuals regardless of age.	Females aged 18–45 years and males aged 18–26 years who have unprotected sex with multiple partners, a history of genital warts, or an abnormal PAP test (female only).
Newfoundland/ Labrador	Females and males who were eligible in Grade 6 are eligible until the person leave the school system (males in Grade 6 are eligible as of September 2017).				
Yukon	Females and male who were eligible in Grade 6 (starting in school year 2011/12 in female and in 2017/18 in male).Females up to 18 years of age at time of first dose.	Males ≤ 26 years.		Individuals ≤ 45 years.	Street involved males ≤ 26 years.
Northwest Territories	Females and males are eligible up to the age of 26.				Females and males up to 26 years of age.
Nunavut	Females and males who would have been in Grade 6 in the 2017/18 school year or later are eligible until Grade 12.				

MSM, men who have sex with men. Sources: Provincial health authorities [12,17,18,29,30,31,32,33,34,35,36].

## Data Availability

Data were extracted from governmental databases and statistics, informal reports, Embase, and PubMed. Data are publicly available.

## Supplementary Materials

The following are available online at https://www.mdpi.com/1718-7729/28/1/97/s1, Table S1: PICO(+) framework.
