# Supplementary material for: Evolution of Public Health Human Papillomavirus Immunization Programs in Canada
Source: Curr Oncol. 2021 Feb 22;28(1):991–1007. doi: 10.3390/curroncol28010097 (PMC7985769; doi:10.3390/curroncol28010097)
Supplement: Supplementary file 1 [file curroncol-28-00097-s001.pdf]

Table S1. PICO(+) framework

Item	Description
Population	All populations that have received an HPV vaccine or have been eligible to receive an HPV vaccine in Canada, by province/territory, including primary and catch-up cohorts and populations at high risk of HPV infection
Interventions	All vaccination programs in Canada
Comparisons	Not applicable
Outcomes	HPV vaccine coverage rate by age, sex, year, and province/territory
Time	No restrictions
Study design	Not applicable
